# Preclinical Activity of the Type II RAF Inhibitor Tovorafenib in Tumor Models Harboring Either a BRAF Fusion or an NF1 Loss-of-Function Mutation

**DOI:** 10.1158/2767-9764.CRC-24-0451

**Published:** 2025-04-23

**Authors:** Shubhra Rastogi, Samantha Perino, Madhu Lal-Nag, Yujin Wang, Samuel C. Blackman, Eleni Venetsanakos

**Affiliations:** Day One Biopharmaceuticals, Inc., Brisbane, California.

## Abstract

Genomic alterations and dysregulation of the MAPK pathway have been described in many different types of cancers. *BRAF*V600 mutations and *BRAF* fusions, found in both pediatric and adult cancers, are oncogenic drivers that drive constitutive activation of the RAF pathway. Neurofibromin 1 (*NF1*) loss-of-function (LOF) mutations, which occur in many cancer types, result in decreased neurofibromin GAP function, thus activating RAS. Tovorafenib is an oral selective, central nervous system–penetrant, type II RAF inhibitor which inhibits RAF monomers and dimers. In this study, the impact of tovorafenib alone or in combination with MEK inhibitor, pimasertib, was explored in adult or pediatric tumor models harboring *BRAF* fusions or *NF1-*LOF mutations. Tovorafenib resulted in tumor regression in an *AGK*::*BRAF* fusion melanoma patient-derived xenograft model *in vivo*, while exhibiting little antitumor activity in *NF1-*LOF tumor models. *In vitro* anti-proliferative activity of tovorafenib and pathway modulation was evaluated in *NF1-*LOF tumor cell lines. Little anti-proliferative activity was observed in the *NF1-*LOF tumor cell lines treated with tovorafenib. In *NF1-*LOF tumor cells treated with tovorafenib, an increase in phosphorylated ERK was observed at low concentrations, with inhibition of phosphorylated ERK at higher concentrations. When tovorafenib was combined with pimasertib *in vitro*, synergy was observed in an *NF1-*LOF embryonal rhabdomyosarcoma patient-derived xenograft model *ex vivo* and an *NF1-*LOF Malignant peripheral nerve sheath tumors (MPNST) cell line *in vitro*, suggesting that vertical pathway inhibition is needed in the *NF1-*LOF mutant setting.

**Significance:**

Tovorafenib demonstrated efficacy in *BRAF* fusion but not in *NF1-*LOF mutant tumor models. Vertical pathway inhibition by combining type II RAF plus MEK inhibitors may have clinical relevance in *NF1-*LOF mutant tumors.

## Introduction

Genomic alterations resulting in dysregulation of the MAPK pathway are known oncogenic drivers in both pediatric and adult cancers that drive constitutive activation of the RAF pathway (1, 2). The family of RAF kinases [BRAF, CRAF (RAF1), and ARAF] consists of the key effectors of RAS that initiate the MAPK signaling cascade. Once activated, RAFs phosphorylate their downstream substrates MEK1/2 which in turn phosphorylate ERK1/2 (3). Activated ERK kinases then phosphorylate other key downstream substrates, for example DUSP6 and SPROUTY, that are critical for cellular processes (4).

Oncogenic *BRAF* alterations that drive constitutive activation of the MAPK pathway include point mutations and gene fusions (1). The most common *BRAF* point mutation is a valine (V) to glutamic acid (E) substitution at BRAF amino acid 600 located in the kinase domain (V600E; ref. 5). The *BRAF *V600E mutation results in a constitutively active monomeric conformation of BRAF in which the kinase domain remains in the active, open configuration allowing RAS-independent monomer signaling (6). Oncogenic BRAF fusions represent a different mechanism of BRAF activation that result from genomic rearrangements. A fusion is a genomic rearrangement that involves fusion of the C-terminal kinase domain of the *BRAF* gene with another gene at the N-terminal position. This genomic rearrangement results in loss of the auto-inhibitory domain of BRAF leading to constitutive activation of the downstream signaling pathway (7, 8). The incidence and type of *BRAF* fusion vary among different cancers. For example, *BRAF* fusions occur in the majority of pediatric low-grade gliomas (pLGG), with the most common being a *KIAA1549*::*BRAF* fusion, present in up to 70% to 80% of pilocytic astrocytomas (9–11). Another example would be the *AGK*::*BRAF* fusion which is the most common BRAF 5′ partner in melanoma and adult lung cancers and present in 19% of pediatric patients with thyroid gland papillary carcinoma (12, 13).

Tovorafenib (also known as DAY101, TAK-580, MLN2480, and BIIB024) is a selective, central nervous system–penetrant, small-molecule type II RAF inhibitor which inhibits *BRAF *V600E mutations as well as both wild-type BRAF and CRAF. Tovorafenib does not result in paradoxical activation of the MAPK signaling in tumors harboring *BRAF* fusions and importantly, has been shown to affect tumors with the *KIAA1549*::*BRAF* fusion (11). Tovorafenib inhibits both RAF monomers and dimers, which is in contrast to type I BRAF inhibitors that selectively inhibit RAF monomers and can induce paradoxical activation of ERK signaling in the presence of wild-type BRAF and activated RAS (1, 9).

Tovorafenib has shown single-agent activity in adult patients with *BRAF*-mutated melanoma in a phase I trial (10). Tovorafenib has shown single-agent activity with meaningful clinical responses and manageable safety profile in children and young adults with *BRAF*-altered relapsed/refractory pLGG in the phase II FIREFLY-1 trial (PNOC026; NCT04775485) resulting in FDA approval in this setting (14–16). Although clinical effectiveness of tovorafenib was demonstrated in pLGGs harboring *BRAF* fusion or *BRAF *V600 mutation, its effectiveness in the treatment of neurofibromin 1 (*NF1*)–associated pLGG remains unknown as these patients were excluded from the FIREFLY-1 trial. The *NF1* loss-of-function (LOF) mutations occur in many cancer types, including pediatric cancers, with 10% to 15% of pediatric patients developing a low-grade glioma within the optic pathway and an additional 3% to 5% patients developing low-grade glioma outside of the optic pathway (17, 18). The *NF1* gene encodes for neurofibromin 1 and *NF1-LOF* results in decreased neurofibromin GAP function, thus activating RAS and downstream MAPK signaling (19, 20). *NF1-*LOF mutations and BRAF alterations are mutually exclusive in pLGG (21, 22). Tovorafenib is also under investigation in combination with the MEK inhibitor, pimasertib, in patients ≥12 years of age with recurrent, progressive, or refractory solid tumors harboring MAPK pathway alterations in the phase Ib/II FIRELIGHT-1 study (NCT04985604; 23).

In this preclinical study, we set out to investigate the impact of tovorafenib alone or in combination with MEK inhibitor pimasertib in adult or pediatric tumor models harboring either an *AGK*::*BRAF* fusion or an *NF1-*LOF mutation.

## Materials and Methods

### Animals

Mice used in *in vivo* experiments were either female NOD/SCID mice (purchased from Vital River Laboratories Research Models and Services) or female BALB/c nude mice (purchased from GemPharmatech, RRID: SCR_017239). All mouse *in vivo* experiments were conducted by Crown Bioscience and in accordance with the animal welfare law (approved by local authorities) and the ethical guidelines of Crown Bioscience. Mice used to generate embryonal rhabdomyosarcoma (ERMS) patient-derived xenograft (PDX) tumors for *ex vivo* use were immunodeficient NOG mice (athymic nude-Foxn1nu; purchased from Taconic Biosciences, RRID: SCR_016410); experiments were conducted by Champions Oncology and performed according to the Institutional Animal Care and Use Committee of Champions Oncology guidelines.

### Tumor-bearing mice and model establishment

Female mice with tumors harboring an *AGK*::*BRAF* fusion melanoma PDX or *NF1-*LOF ERMS PDX were established by inoculating NOD or NOG/SCID mice, respectively, with tumor fragments harvested from stock mice. Mice with tumors harboring an *NF1-*LOF mutation MeWo xenograft were established in 10 mice per treatment group by inoculating 7- to 9-week-old female BALB/c nude mice on the right flank with tumor cells (5 × 10^6^) in 0.1 mL of PBS mixed with Matrigel (1:1) for tumor development. For the *AGK*::*BRAF* fusion melanoma PDX model, 7- to 8-week-old mice were inoculated subcutaneously on the right flank and randomized into eight mice per treatment group. For the *NF1-*LOF ERMS PDX model, tumor fragments were harvested from stock animals and implanted unilaterally on the left flank of 6- to 8-week-old mice and then the mice were randomized into 10 mice per treatment group. After tumor cell inoculation, the animals were checked daily for morbidity and mortality. All experiments were performed once.

For *ex vivo* experiments, NOG mice were implanted with ERMS PDX tumor fragments and harvested for growth *ex vivo* in cell culture (Supplementary Table S1).

### Suspension vehicle and tovorafenib preparation—*in vivo* experiments

Purified water (type II or high-performance liquid chromatography grade) was used as aqueous suspension vehicle and control for *in vivo* efficacy experiments. Amorphous tovorafenib spray-dried dispersion material (Serán BioScience) was prepared by suspending into a vehicle to achieve a final aqueous suspension of 5 mg/mL. Tovorafenib was freshly prepared every day prior to dosing.

### Antitumor efficacy *in vivo*

The antitumor activity of tovorafenib was assessed as a single agent *in vivo* using the established murine tumor models harboring either the *BRAF* fusion or an *NF1-*LOF mutation. Mice were randomized to either control (vehicle) or a tovorafenib dose group when their mean tumor size reached 100 to 300 mm^3^. Randomization was performed based on the “Matched distribution” method (StudyDirector software, version 3.1.399.19) with the date of randomization denoted as day 0. Tumor volumes and body weight were measured twice per week after randomization. Tumor volume was measured in two dimensions (2D) using a caliper, and the volume was expressed in mm^3^ using the formula V = (L × W × W)/2, in which V was tumor volume, L was tumor length (the longest tumor dimension), and W was tumor width (the longest tumor dimension perpendicular to L). Percentage change in tumor volume and body weight was calculated using StudyDirector software (version 3.1.399.19).

For the *AGK*::*BRAF* fusion model, tovorafenib was given orally daily at clinically relevant doses of either 17.5 or 25 mg/kg for 14 days. In order to be deemed clinically relevant, Trough concentration (Ctrough) and daily AUC values for 17.5 and 25 mg/kg in mice were comparable with once weekly 400 and 600 mg doses, respectively, in humans. For the *NF1-*LOF models, 25 mg/kg tovorafenib was given orally daily for either 21 or 28 days. The mice in the control vehicle group were dosed either daily or twice daily.

### Phosphorylated ERK modulation *in vivo*—pharmacokinetic–pharmacodynamic study

Modulation of phosphorylated ERK (pERK) levels by tovorafenib as a single agent in the *AGK*::*BRAF* fusion and *NF1-*LOF melanoma models was assessed by Crown Bioscience. *AGK*::*BRAF* fusion tumor–bearing mice were randomized to a single dose orally of tovorafenib, 17.5 or 25 mg/kg, or the vehicle control when tumor volume reached 300 to 500 mm^3^. Tumor and plasma samples were collected at 4, 8, and 24 hours after dose administration (three mice per timepoint). *NF1-*LOF tumor–bearing mice were randomized to a single dose of 25 mg/kg tovorafenib or the vehicle control when tumor volume reached 300 to 500 mm^3^. Tumor and plasma samples were collected at 4 and 24 hours after dose administration (three mice per time point). Tumor samples were snap-frozen and analyzed by Western blotting for pERK levels. Plasma samples were analyzed by the LC/MS-MS method to confirm plasma drug concentration.

### Western blotting

Tumor samples were lysed with RIPA buffer (Thermo Fisher Scientific, cat. no. 89901) containing one complete EDTA-free tablet (Roche, cat. no. 05892791001) and one phosphatase inhibitor cocktail tablet (Roche, cat. no. 04906845001) per 10 mL of RIPA buffer. Protein concentration was determined using bicinchoninic acid assay (Pierce, cat. no. 23227). Samples were loaded at 25 to 30 μg/lane on 4% to 12% NuPAGE Bis-Tris gels (Invitrogen, cat. no. WG1403BOX) and were run in MOPS-SDS running buffer (Invitrogen, cat. no. NP0001) at constant voltage (120V) for approximately 2 hours. The gels were transferred onto a pre-activated polyvinylidene fluoride membrane (Millipore, cat. no. IPVH00010) in transfer buffer (Bio-Rad, cat. no. 161-0734). The membrane was blocked with 5% BSA (Sigma, cat. no. A7030) in TBS/0.1% Tween 20 for 1 hour at room temperature. The membrane was incubated overnight at 4°C with primary antibody in 0.5% BSA in TBS/0.1 Tween-20. The primary antibodies used were phospho-p44/42 MAPK ERK1/2 (T202/Y204; rabbit; Cell Signaling Technology, cat. no. 4370, RRID: AB_2315112, 1:2,000) to measure pERK and p44/42 MAPK ERK1/2 (rabbit; Cell Signaling Technology, cat. no. 4695, RRID: AB_390779, 1:1,000) to measure total ERK and anti-GAPDH (mouse; Kangchen Biotech, cat. no. KC-5G4, RRID: AB_2493106; diluted 1:10,000) as a loading control. The membrane was incubated with horseradish peroxidase–conjugated anti-rabbit antibody (Cell Signaling Technology, cat. no. 7074s, RRID: AB_2099233, 1:1,000) and IRDye 680RD goat anti–mouse immunoglobulin G (LI-COR Biosciences, cat. no. 926-68070, RRID: AB_10956588, 1:5,000) for 1 hour at room temperature. Blots were scanned and quantified using the Odyssey (LI-COR Biosciences) or Tanon 5200 Multi Image system (Tanon), and each sample was normalized to allow comparison.

### Tumor cell line models

Tumor cell lines used for *in vitro* experiments are outlined in Supplementary Table S1. In brief, these included sNF96.2 (*NF1-*LOF), MeWo (*NF1-*LOF), NCI-H1838 (*NF1-*LOF), and A375 (*BRAF *V600E) purchased from ATCC.

Unless otherwise stated, *in vitro* experiments were conducted by WuXi AppTec, RRID: SCR_001217. Additionally, an ERMS PDX (*NF1-*LOF) tumor model, generated by Champions Oncology, was used for *ex vivo* experiments. Culture media DMEM and RPMI-1640 were purchased from Gibco (cat. no. 11965-092 and 22400-089, respectively), and Eagle Minimum Essential Medium was purchased from ATCC (cat. no. 30-2003).

### Test compound preparation—*in vitro* experiments

For use in Meso Scale Discovery (MSD) ELISA and cell viability assays *in vitro*, tovorafenib, TAK-632, vemurafenib, LXH254, BGB-283, and belvarafenib were tested in duplicate in sNF96.2, MeWo, and A375 cell lines and in singlicate with NCI-H1838 at a range of concentrations from 10 μmol/L. An eight-step three-fold serial dilution of stock compound in 100% DMSO was conducted to create a 1,000× stock for each compound. Next, 2 μL of each concentration was added to 198 μL assay medium (PBS) to create a 10× stock. From here, the compounds were added to a prepared 96-well assay plate containing cells (sNF96.2, MeWo, A375, or NCI-H1838) to yield a final concentration range of 10 μmol/L to 1.52 nmol/L and a final DMSO concentration of 0.1% for all cell viability and ELISA experiments. MSD ELISA experiments were conducted by WuXi AppTec on whole cell lysates and generated using the Phospho/Total ERK1/2 Assay Whole Cell Lysate Kit (MSD, cat. no. K15107D) according to the manufacturer’s protocol.

TAK-632, LXH254, and belvarafenib are type II RAF inhibitors and were included as positive controls (see Supplementary Table S3). Vemurafenib was included as a type I BRAF inhibitor control. BGB-283 is a dual RAF and EGFR inhibitor and included as an additional control. For the vehicle control group, 0.1% DMSO in assay medium was used.

### pERK modulation *in vitro*

*In vitro* modulation of pERK levels was assessed in *NF1-*LOF and *BRAF *V600E tumor cell lines by MSD ELISA. Cell concentrations were adjusted for cell density (seeding density of 40,000 for sNF96.2, MeWo, and A375; seeding density of 20,000 for NCI-H1838) prior to plating onto a 96-well assay plate and incubated overnight at 37°C with 5% CO_2_, 95% air, and 100% relative humidity.

pERK levels were assessed 1 hour after treatment with each of the test compounds or control. Preparation of 96-well plates for MSD ELISA was performed according to the manufacturer’s instructions using the Phospho/Total ERK1/2 Assay Whole Cell Lysate Kit (MSD, cat no. K15107D). The plates were read using the MSD reader (MESO QuickPlex SQ120, cat. no. SQ120, RRID: SCR_020304). The A375 *BRAF *V600E tumor cell line served as a positive control.

In addition, the difference in kinetics of pERK modulation in *NF1-*LOF tumor cell lines was assessed by measuring pERK levels at 1 and 6 hours after treatment with tovorafenib in sNF96, MeWo, and NCI-H1838 cells.

The percent phosphoprotein in a sample was calculated using MSD phospho-/total multiplex phosphoprotein assays, as well as the following formula: anti-total and anti-phospho assay in the same well: % phosphoprotein = [(2 *×* phospho signal)/(phospho signal + total signal)] × 100. The ratio of pERK to total ERK was normalized to the ratio of the DMSO control group. EC_50_ pERK values and concentration response curves were generated using GraphPad Prism software analysis (RRID: SCR_002798).

### Cell viability assay

The *in vitro* anti-proliferation effect of each of the test compounds was assessed in *NF1-*LOF and *BRAF *V600E tumor cell lines cultured as a monolayer using the CellTiter-Glo (CTG) cell viability assay. Cell culture experiments were performed with duplicate wells. Cell concentrations were adjusted for cell density (seeding density of 5,000 for sNF96.2 and MeWo; 6,000 for NCI-H1838; and 2,500 for A375) prior to plating onto a 96-well assay plate and incubated overnight at 37°C with 5% CO_2_, 95% air, and 100% relative humidity.

Anti-proliferative activity was assessed after 72 hours of treatment with the test compound or control. Daily repeated application of tovorafenib was necessary to maintain drug concentration during the 72-hour period because of the tendency of tovorafenib to adhere to plastic and FBS over time when in solution. DMSO was also applied as a control on days 2 and 3. The final DMSO concentration for all test compounds and vehicle control was 0.1%. The A375 *BRAF *V600E tumor cell line served as a positive control. Preparation of 96-well plates for CTG assays was performed according to the manufacturer’s instructions using the CTG Luminescent Cell Viability Assay Kit (Promega, RRID: SCR_006724, cat. no. G7573). Luminescence was measured on day 3 using the 2104 EnVision Multilabel Reader (PerkinElmer).

The inhibition rate of the tested compounds was determined using the following formula: inhibition rate (%) = (1 − [relative luminescence unit (RLU) compound − RLU blank])/(RLU control − RLU blank) × 100. EC_50_ proliferation values were generated using GraphPad Prism software analysis.

### Time-resolved fluorescence energy transfer binding assay

Tovorafenib inhibition of ARAF was assessed by time-resolved fluorescence energy transfer binding assay and conducted by Enzymlogic using their KINETICfinder platform to determine IC_50_ values. Tovorafenib (GNR-#104-1606-009) and TAK-632 (GNR-#104-1606-020) were prepared using a 10-point serial dilution in 100% DMSO at 100× final assay concentration. Compounds were mixed with assay buffer (50 mmol/L HEPES pH 7.5, 1 mmol/L Ethyleneglycol-bis-(aminoethyl-ether)-N,N,N',N'-tetraacetic acid, 0.01% Brij-35, 10 mmol/L MgCl_2_, and 2 mmol/L Dithiothreitol), and 5 μL of this solution was transferred to the assay plate. The final concentration of DMSO in the assay was 1%. Hundred percent DMSO was used as a high and low control. Assays were run in singlicate with a reference compound included in each assay to evaluate reproducibility and assay quality.

Wells containing the fluorescent probe, antibody, and target (ARAF) were used as the high reaction control (total binding) and wells containing the probe and antibody were used as the low reaction control (nonspecific binding). Tovorafenib or TAK-632 was mixed with 5 nmol/L active recombinant ARAF (partial length construct, derived from human wild-type). Next, the substrate antibody solution was added, and the reaction was incubated for 60 minutes at room temperature. Percentage inhibition of each well was calculated using the following formula: % inhibition = 100 − [compound emission ratio (ER) − low control ER/high control ER − low control ER] × 100. Results were fitted to a concentration response curve with a variable slope and constraints of 0 and 100 for bottom and top, respectively, to generate the IC_50_ values.

### Combination studies

#### sNF96.2 cell line—2D monolayer assay

The cytotoxicity of tovorafenib (MA 18-004) plus pimasertib (PGS-1156a) was assessed *in vitro* using the sNF96.2 cell line. These experiments were performed and analyzed by Charles River Laboratories (RRID: SCR_003792). Synergy of this combination was assessed using a 5 × 5 matrix combination in a 2D monolayer assay 72 hours after treatment with a concentration range of 10 to 0.01 μmol/L for each compound. For tovorafenib, repeated application occurred on days 2 and 3 due to the aforementioned tendency when in solution for long periods of time. Pimasertib was only added once at the start of the experiment. Cell viability was determined by CTG assay.

Synergy scores were determined using the SynergyFinder web application (available at https://synergyfinder.fimm.fi/) using the Loewe additivity model, which calculates the expected response if two drugs are expected to produce the same effect. Any score ≥10 suggests a synergistic interaction between the two drugs; scores ≤10 indicate antagonism (24, 25).

#### ERMS PDX model—tumor fragment assay

The cytotoxicity of TAK-632 plus pimasertib was assessed using ERMS PDX model tumor cells isolated from the PDX tumor fragments and cultured *ex vivo*. These experiments were performed and analyzed by Champions Oncology. Tumor fragments (∼500–1,500 mm^3^) were excised and placed in MACS media with antibiotic and antimycotic on ice until dissociated into fragments using enzymatic digestion. Large fragments were removed by filtration (using a 500-μm filter followed by a 200-μm filter). The remaining fragments were suspended in PDX media (Supplementary Table S1). Tumor cells were seeded at a density of 1,000 cells/40 μL per well in PDX media. Synergy of TAK-632 plus pimasertib was evaluated using a 6 × 6 matrix combination in three dimensions (3D) 144 hours after treatment with a concentration range of 10 to 0.04 μmol/L for each compound. TAK-632 and pimasertib were added once at the start of the experiment. The 3D CTG assay (Promega, RRID: SCR_006724, cat. no. G9682) was used to quantify cell viability. Due to the 3D format of the assay, conditions were not amenable for repeated application of tovorafenib; thus, TAK-632 was used as a positive type II RAF inhibitor control. Assays were run in quadruplicate, and 0.1% DMSO and 10% DMSO served as a vehicle and positive control, respectively.

As above, synergy scores were determined using the SynergyFinder web application (available at https://synergyfinder.fimm.fi/) using the Loewe additivity model. Any score ≥10 suggests a synergistic interaction between the two drugs; scores ≤10 indicate antagonism (24). Analysis of the drug combination for synergy was performed using Lumin Bioinformatics software (Champions Oncology).

### Data availability

The data generated in this study are not publicly available because of them being confidential, proprietary company-owned data but are available upon reasonable request from the corresponding author.

## Results

### Tovorafenib is effective in *AGK*::*BRAF* fusion melanoma PDX *in vivo*

**Figure 1:**
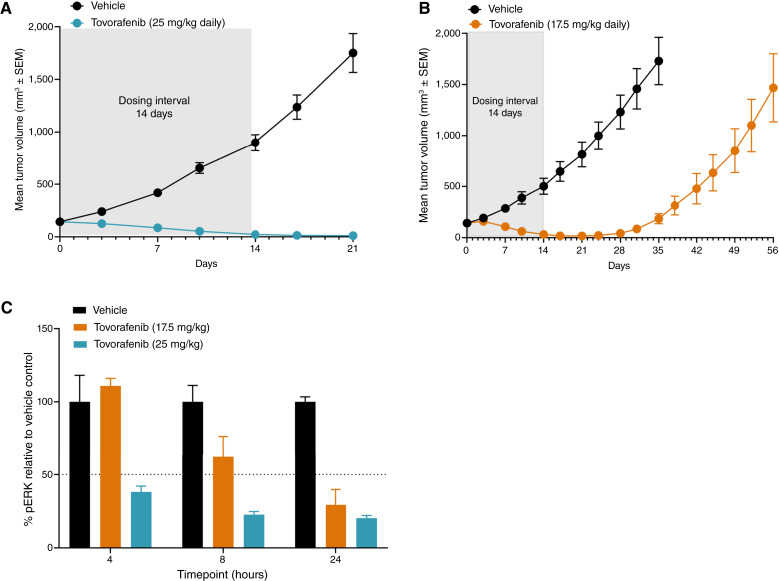
Tovorafenib in the *AGK*::*BRAF* fusion melanoma PDX model *in vivo*. Female NOD/SCID mice bearing *AGK*::*BRAF* fusion melanoma PDX tumors were dosed orally once-daily for 14 days with tovorafenib at either (**A**) 25 or (**B**) 17.5 mg/kg or vehicle. Tumor volume and body weight were measured twice weekly. **C,** Female NOD/SCID mice with tumors harboring the *AGK*::*BRAF* fusion were given a single oral dose of tovorafenib (25 or 17.5 mg/kg) or vehicle. Tumors and plasma were collected at 4, 8, and 24 hours post-dose. Tumors were snap-frozen and analyzed for pERK levels using Western blots. Graphs were generated using GraphPad Prism software analysis.

The effect of tovorafenib on tumor growth was assessed in an *in vivo* efficacy study. In mice bearing melanoma PDX tumors harboring an *AGK*::*BRAF* fusion, tumor regression was observed in response to oral administration of tovorafenib for 14 days at dosage levels of 25 and 17.5 mg/kg daily assessed in two separate efficacy studies, respectively (Fig. 1A and B, respectively). Both dosage levels in both studies have exposures and Ctrough that are clinically relevant (see Supplementary Table S2; Supplementary Fig. S2). Experiments involving mice treated at the 25 mg/kg dose were terminated at day 21 because similar levels of regression were observed as in the 17.5 mg/kg dose with no additional benefit expected. Tovorafenib was well tolerated at both dose levels in both studies, with no significant body weight loss observed in either dose level throughout the treatment (Supplementary Fig. S1). In the two separate efficacy studies, tumor regression was sustained for at least 7 days after the last dose given on day 14. On day 28, approximately 2 weeks after the last dosage of 17.5 mg/kg daily, tumors started to regrow.

In a single-dose pharmacokinetic–pharmacodynamic (PK–PD) study, mice bearing *AGK*::*BRAF* fusion melanoma PDX tumors were orally administered tovorafenib at 17.5 or 25 mg/kg. Tumors were harvested at various timepoints after dose to assess pERK. Inhibition of pERK was observed 8 and 24 hours after dose in response to 17.5 mg/kg tovorafenib and at 4, 8, and 24 hours after dose in response to 25 mg/kg tovorafenib compared with the vehicle control (Fig. 1C).

### Tovorafenib is ineffective in *NF1-*LOF models *in vivo*

**Figure 2:**
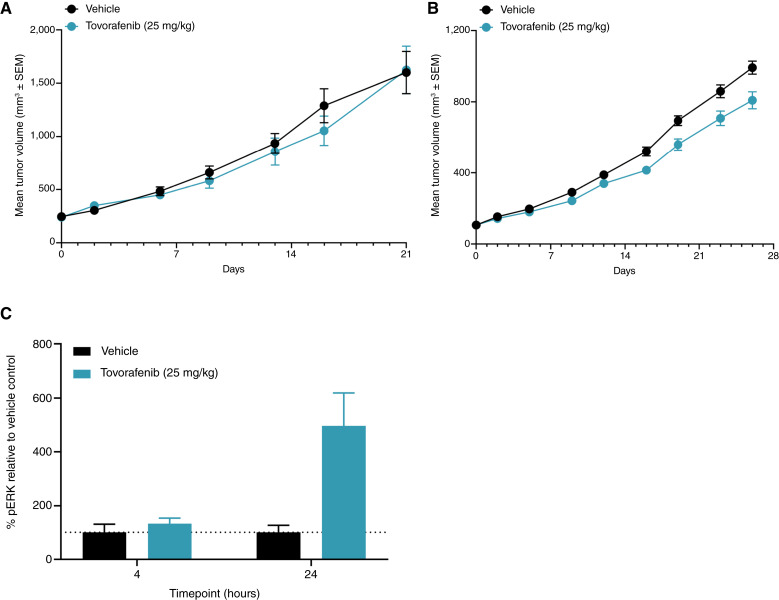
Tovorafenib in *NF1-*LOF tumor models *in vivo*. Female mice bearing either (**A**) *NF1-*LOF ERMS PDX or (**B**) *NF1-*LOF MeWo xenograft tumors were dosed orally once-daily for 21 or 28 days, respectively, with tovorafenib (25 mg/kg) or vehicle. The control vehicle group was dosed twice daily. Tumor volume and body weight were measured twice weekly. **C,** Female BALB/c nude mice with tumors harboring an *NF1-*LOF mutation (MeWo) were given a single oral dose of tovorafenib (25 mg/kg) or vehicle. Tumors and plasma were collected at 4 and 24 hours post-dose. Tumors were snap-frozen and analyzed for pERK levels using Western blots. Graphs were generated using GraphPad Prism software analysis.

The impact of tovorafenib on tumor growth was assessed in two different *NF1-*LOF mutant models, including an *NF1-*LOF mutant ERMS PDX model and an *NF1-*LOF melanoma xenograft tumor model, MeWo. Lack of significant antitumor activity was observed in the ERMS PDX and MeWo models following treatment with tovorafenib at 25 mg/kg orally daily for 21 and 28 days, respectively (Fig. 2A and B, respectively). Tovorafenib was well tolerated in both models. In addition, a single-dose PK–PD study using the MeWo melanoma xenograft model was conducted with tovorafenib at 25 mg/kg. No change in pERK was observed 4 hours after tovorafenib dose; however, an increase was observed 24 hours after dose relative to the vehicle control (Fig. 2C).

### pERK modulation and proliferation in *NF1-*LOF and *BRAF *V600E tumor cell lines *in vitro*

**Figure 3:**
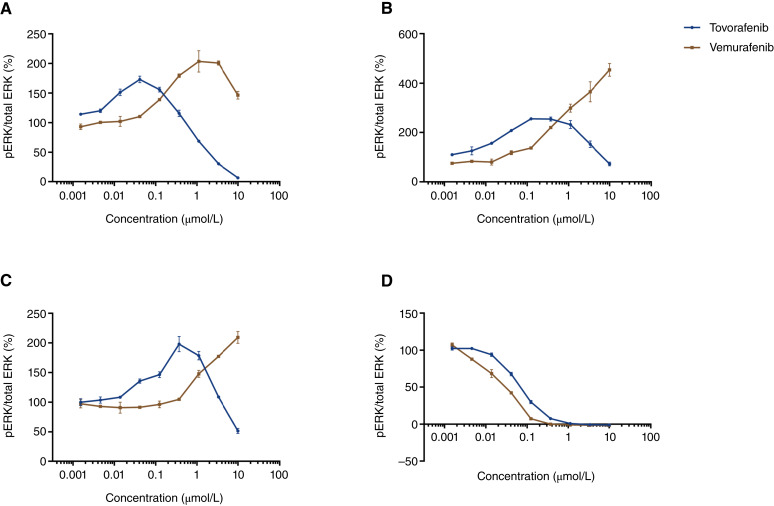
Concentration–response curves of pERK modulation in *NF1-*LOF and *BRAF *V600E tumor cell lines. pERK levels were measured using MSD ELISA 1 hour after treatment with either tovorafenib or vemurafenib in tumor cell lines harboring either an *NF1-*LOF mutation: (**A**) sNF96.2, (**B**) MeWo, or (**C**) NCI-H1838 or a *BRAF *V600E mutation: (**D**) A375. Graphs represent the normalized fold of the ratio of pERK to total ERK expressed as a percentage. All experiments were performed twice except with NCI-H1838 which was performed once. Concentration–response curves were generated using GraphPad Prism software analysis.

The levels of pERK were measured using MSD ELISA 1 hour after treatment in three *NF1-*LOF tumor cell lines, including sNF96.2, MeWo, and NCI-H1838. Increased pERK levels were observed at lower concentrations of tovorafenib and decreased pERK levels at higher tovorafenib concentrations (Fig. 3A–C). Vemurafenib served as a type I BRAF inhibitor control and as expected, pERK levels increased with vemurafenib concentration in all *NF1-*LOF tumor cell lines (Fig. 3A–C). The peak of the pERK increase observed with tovorafenib occurred at a lower concentration and did not reach the level observed with vemurafenib, which showed a continual increase in pERK with increasing concentration. The A375 *BRAF *V600E cell line served as a positive control for pERK inhibition by both tovorafenib and vemurafenib (Fig. 3D).

The anti-proliferative activity of tovorafenib and TAK-632 was evaluated by CTG cell viability assay at 72 hours after treatment in the *NF1-*LOF tumor cell lines and the *BRAF *V600E tumor cell line, A375. Tovorafenib was potent in the A375 cell line, consistent with historical data (11). However, little activity was observed in the *NF1-*LOF tumor cell lines, MeWo and NCI-H1838 (Table 1; Supplementary Fig. S3). The *NF1-*LOF MPNST tumor cell line, sNF96.2, was sensitive to tovorafenib with a proliferation EC_50_ of 0.99 μmol/L, which was comparable with the A375 EC_50_ proliferation. All three *NF1-*LOF tumor cell lines were insensitive to vemurafenib (Table 1).

**Table 1:** EC_50_ for pERK and proliferation across tumor cell lines

Cell line	Mutation	Tumor type	pERK EC_50_ μmol/L			Proliferation EC_50_ μmol/L		
			Tovorafenib	TAK-632	Vemurafenib	Tovorafenib	TAK-632	Vemurafenib
sNF96.2	NF1 p.Asn1229Metfs*11(c.3683delC)	MPNST	1.6	0.75	>10	0.99	2.4	6
MeWo	*NF1* ^Q336^	Melanoma	8.3	1.6	>10	7.5	4.1	4.5
NCI-H1838^a^	*NF1* ^184fs^	Lung	>10	1.25	>10	>10	3.3	>10
A375	*BRAF *V600E	Melanoma	0.07	0.03	0.02	0.62	0.07	0.09

The number of experiments for sNF96.2, MeWo, and A375 is 2.

a For NCI-H1838, *n* = 1. Cell viability was assessed using CTG 72 hours after treatment. Repeated application of tovorafenib was done in CTG assay. pERK levels were measured using MSD ELISA 1 hour after treatment. pERK and proliferation EC_50_ values were determined using GraphPad Prism software analysis.

**Figure 4:**
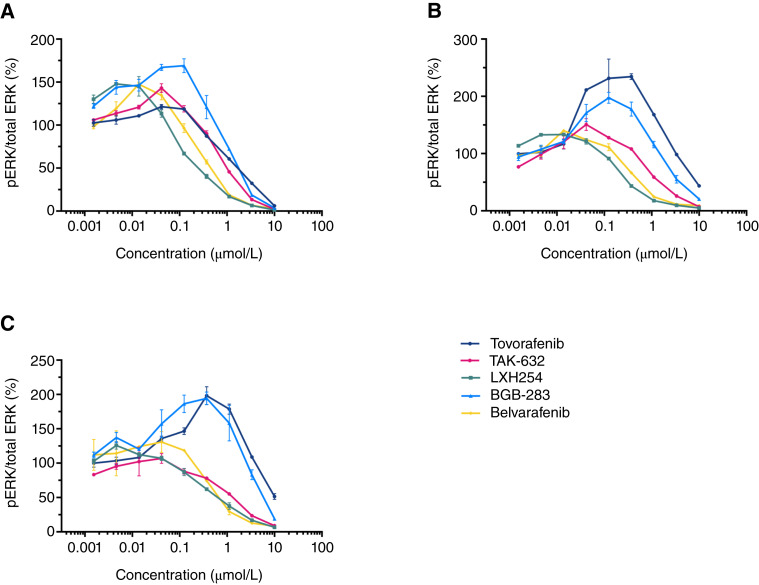
pERK modulation in response to tovorafenib, TAK-632, LXH254, BGB-283, and belvarafenib in *NF1-*LOF tumor cell lines. pERK levels were measured using MSD ELISA 1 hour after treatment with either tovorafenib, TAK-632, LXH254, BGB-283, or belvarafenib in tumor cell lines harboring an *NF1-*LOF mutation: (**A**) sNF96.2, (**B**) MeWo, and (**C**) NCI-H1838. Graphs represent the normalized fold of the ratio of pERK to total ERK expressed as a percentage. These experiments were performed once. Concentration–response curves were generated using GraphPad Prism software analysis.

To further explore the pERK modulation pattern exhibited by tovorafenib, additional type II RAF inhibitors were assessed, including TAK-632, LXH254, BGB-283, and belvarafenib. All these type II RAF inhibitors inhibited pERK as expected. Similar to tovorafenib, increased pERK levels were observed at lower concentrations and decreased pERK at higher concentrations with each type II RAF inhibitors in all three *NF1-*LOF tumor cell lines at the 1-hour timepoint (Fig. 4A–C). In MeWo and NCI-H1838, there were some differences in the concentration at which the pERK peak occurred across the type II RAF inhibitors. In MeWo, the pERK peak occurred at a higher concentration when compared with LXH254, belvarafenib, and TAK-632. In NCI-H1838, the pERK increase was to a higher extent for tovorafenib and BGB-283 compared with a lesser extent of the pERK increase for LXH254, TAK-632, and belvarafenib. Overall, these data showed a bell-shaped curve of pERK modulation in response to tovorafenib, TAK-632, LXH254, BGB-283, and belvarafenib in *NF1-*LOF mutant tumor cell lines, suggesting that this may potentially be a class effect.

**Figure 5:**
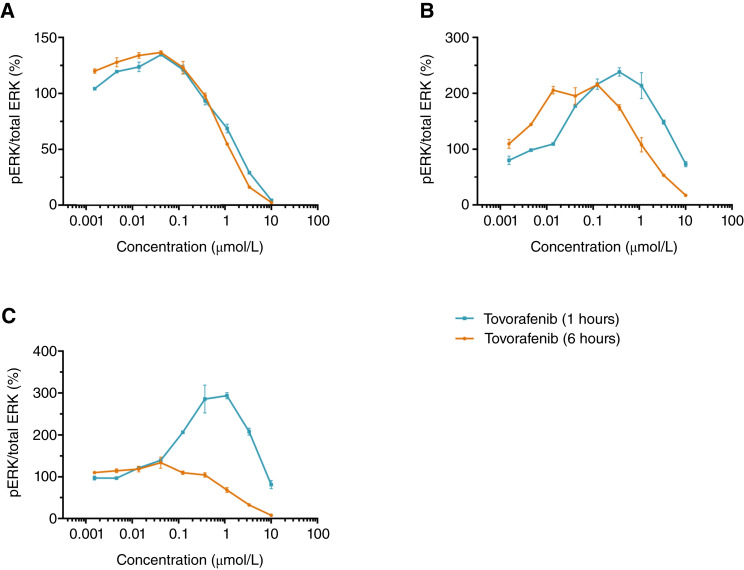
Kinetics of pERK modulation in *NF1-*LOF tumor cell lines with tovorafenib. pERK levels were measured using MSD ELISA 1 and 6 hours after treatment in tumor cell lines harboring an *NF1-*LOF mutation: (**A**) sNF96.2, (**B**) MeWo, and (**C**) NCI-H1838. Graphs represent the normalized fold of the ratio of pERK to total ERK expressed as a percentage. These experiments were performed once. Concentration–response curves were generated using GraphPad Prism software analysis.

The kinetics of pERK modulation was also investigated with tovorafenib in the *NF1-*LOF tumor cell lines. Levels of pERK after treatment with tovorafenib at 1 versus 6 hours were also evaluated by MSD ELISA to determine any potential differences in the kinetics of pERK modulation. A similar pattern to Fig. 3A–C was observed after 6 hours, with increased pERK levels at lower concentrations of tovorafenib and pERK decreasing at higher inhibitor concentrations (Fig. 5A–C). Little difference was observed in the concentration response curves in sNF96.2 or MeWo cells at 1 versus 6 hours (Fig. 5A and B). In contrast, in the NCI-H1838 cell line, there was a lack of increase in pERK for tovorafenib across the full concentration range at 6 hours (Fig. 5C).

### Tovorafenib is ARAF sparing *in vitro*

Tovorafenib and TAK-632 IC_50_ values against ARAF were determined via time-resolved fluorescence energy transfer assay. The IC_50_ of tovorafenib was calculated as 55 nmol/L. The shift in the IC_50_ of ARAF relative to wild-type BRAF and CRAF was 5.5× and 78×, respectively. This suggests that tovorafenib does not significantly inhibit ARAF (Table 2). The ARAF IC_50_ for TAK-632 was 215 nmol/L (Table 2; refs. 26–28). For TAK-632, the shift in the IC_50_ of ARAF relative to wild-type BRAF and CRAF was 26× and 52×, respectively.

**Table 2:** Tovorafenib: biochemical IC_50_ data

Compound	IC_50_ nmol/L			
	BRAF wt	CRAF wt	*BRAF *V600E	ARAF
Tovorafenib	10.1	0.7	7.1	55
TAK-632	8.3	1.4	ND	215

IC_50_ values for tovorafenib inhibition of BRAF wild type, CRAF wild type, and *BRAF *V600E (full length, human-derived constructs) were taken from refs. 27, 40, 41. In brief, assay conditions for these constructs were 0.125 nmol/L of GST-tagged BRAF wild type and 12.5 nmol/L of biotinylated MEK; 0.07 5 nmol/L of GST-tagged CRAF wild type and 50 nmol/L of biotinylated MEK; or 0.125 nmol/L of GST-tagged *BRAF *V600E and 100 nmol/L of biotinylated MEK. The ATP concentration in these assays was adjusted between 10 and 50 μmol/L, depending on the Kaplan–Meier of the RAF isoform being assessed (50 μmol/L for BRAF wild type, 10 μmol/L for CRAF wild type, or 25 μmol/L for *BRAF *V600E). The ARAF construct was a partial length, active recombinant human-derived tested at 5 nmol/L.

Abbreviations: Wt, wild type.

### Synergy was observed with type II RAF inhibitors combined with pimasertib in *NF1-*LOF tumor models *in vitro* or *ex vivo*

**Figure 6:**
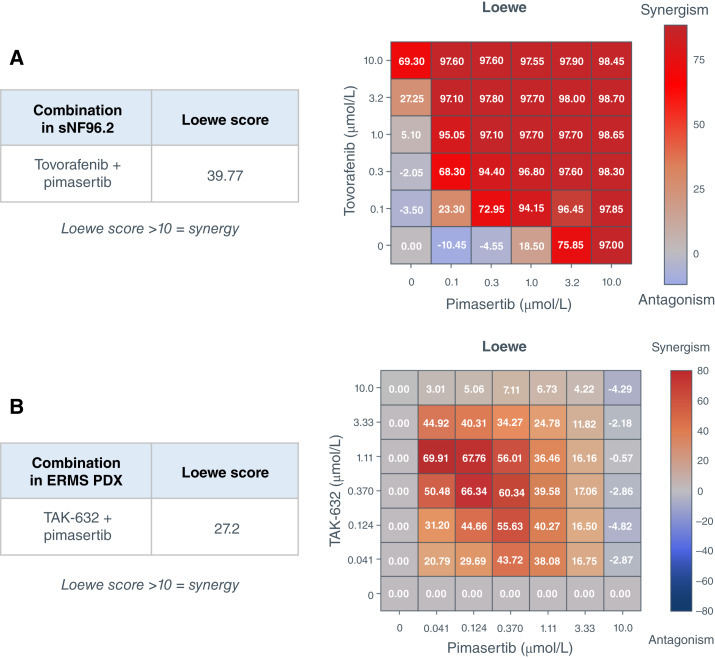
Combination of type II RAF inhibitors plus pimasertib in *NF1-*LOF tumor models. **A,** In sNF96.2 cells, synergy was assessed 72 hours after treatment with tovorafenib plus pimasertib in a 5 × 5 matrix combination format in 2D, followed by Loewe analysis using SynergyFinder 3.0. Pimasertib was added once at the start of the experiment, and for tovorafenib, repeated application occurred on days 2 and 3. Positive values (Loewe score >10, red) indicate synergy, negative values (Loewe ≤ −10, blue) indicate antagonism, and −10 to 10 is neutral indicating an additive effect. **B,** ERMS PDX model tumor cells were isolated from the PDX tumor fragments and cultured *ex vivo* to assess synergy after 144 hours of treatment with TAK-632 plus pimasertib in a 6 × 6 matrix combination format in 3D, followed by Loewe analysis using SynergyFinder 3.0. Positive values (Loewe score >10, red) indicate synergy, negative values (Loewe score <10, blue) indicate antagonism, and Loewe score between −10 and 10 indicate an additive effect. These experiments were performed once in single wells for the sNF96.2 cells and quadruplicate wells for the ERMS PDX model tumor cells.

The combination of tovorafenib plus pimasertib was evaluated *in vitro* using sNF96.2 at 72 hours after treatment (Fig. 6A). The combination of TAK-632 plus pimasertib was evaluated *ex vivo* in the ERMS PDX model (Fig. 6B). Two distinct methodologies were used to generate the analysis of the drug combination for synergy in each of the *NF1-*LOF model (see “Materials and Methods” for details). Both methodologies used SynergyFinder to calculate the Loewe synergy scores. Synergy was observed with pimasertib in combination with either tovorafenib or TAK-632 in both the *NF1-*LOF models with Loewe scores of 39.8 and 27.2 in the sNF96.2 cell line and *ex vivo* ERMS PDX model, respectively, and Bliss scores of 19.0 and 29.8, respectively (Fig. 6; Supplementary Fig. S4). The data indicate that the combination of a type II RAF inhibitor and MEK inhibitor may affect the growth of tumors harboring an *NF1-*LOF mutation.

## Discussion

Tovorafenib is the first and only FDA-approved type II RAF inhibitor for patients with relapsed or refractory pLGG harboring a BRAF fusion or rearrangement or *BRAF *V600 mutation. Tovorafenib has demonstrated clinically meaningful responses and a manageable safety profile in patients with pLGG with *BRAF* alterations including the *KIAA1549*::*BRAF* fusion and *BRAF *V600E mutations (14). However, the clinical effectiveness of tovorafenib in treatment for *NF1*-associated pLGG remains to be addressed as patients with *NF1-*LOF mutations were excluded from the FIREFLY-1 trial. In pLGG, *NF1-*LOF mutations and *BRAF* alterations are mutually exclusive.

The impact of tovorafenib on *in vivo* antitumor activity was assessed in an *AGK*::*BRAF* fusion melanoma PDX model and in two *NF1-*LOF models, including an ERMS PDX and the MeWo melanoma xenograft. As expected, based on published activity in *BRAF* fusion models, daily administration of tovorafenib at clinically relevant doses resulted in tumor regression in the *AGK*::*BRAF* fusion melanoma model. Notably, durable regressions were observed with no tumor regrowth for approximately 2 weeks after the last administered dose of tovorafenib. Taken together, the preclinical efficacy in the *AGK*::*BRAF* fusion melanoma model, and the fact that tovorafenib has been approved for relapsed/refractory pLGGs, supports tissue agnostic activity for tovorafenib in tumors harboring *BRAF* fusions or rearrangements.

In both *NF1-*LOF *in vivo* tumor models, there was no antitumor activity observed with tovorafenib at clinically relevant exposures. The data are consistent with lack of tovorafenib antitumor activity being reported in granulocytes/erythroids/macrophages/megakaryocytes (GEMM) models of plexiform neurofibroma harboring germline *NF1-*LOF mutations (29). Furthermore, no exacerbated tumor growth was observed in the tovorafenib-treated group relative to the vehicle group in our efficacy study. This is in contrast with the observation in the GEMM model in which a qualitative difference was observed in nine of 48 proximal nerve volume measurements in the tovorafenib-treated group which were relatively larger, (i.e., >3 mm^3^). The tovorafenib plasma exposure was comparable between the two studies. Therefore, discrepancies in data observed between the two studies could be attributed to the differences in tissue or disease type and/or the specific *NF1* mutation.

In the single-dose PK–PD study with tovorafenib in the *NF1-*LOF tumor model, a pERK increase was only observed at 24 hours and not at earlier time points. This suggests that the increase in pERK observed is likely dependent on tovorafenib plasma exposure. When administered daily at clinically relevant exposures, the increase in pERK levels may be a transient response and therefore may not be sustained for a long duration to drive exacerbated tumor growth in preclinical *NF1-*LOF tumor models. Higher doses were not explored as our studies aimed to remain clinically relevant. To understand the differences in pERK modulation in response to tovorafenib in the *BRAF* fusion versus *NF1-*LOF setting, we further evaluated pERK modulation in three *NF1-*LOF tumor cell lines *in vitro*. Consistent with *in vivo* PD results, increased pERK was observed in all three cell lines at lower concentrations of tovorafenib, whereas pERK inhibition was evident at higher concentrations. This bell-shaped curve of pERK modulation was different to that observed with vemurafenib, a type I BRAF inhibitor that causes paradoxical activation of the MAPK pathway in cells expressing wild-type BRAF in the presence of activated RAS (9, 30). As *NF1-*LOF mutations are known to activate RAS signaling (31), it is not surprising that vemurafenib would result in paradoxical activation of pERK, which increases with dose in this setting as well. In contrast, increased pERK levels were observed at lower concentrations of tovorafenib and decreased pERK at higher concentrations of tovorafenib. It was also interesting that pERK levels at lower concentrations of tovorafenib were below the levels observed with vemurafenib. The kinetics of the pERK increase at lower concentrations differed across the *NF1-*LOF tumor cell lines. The bell-shaped curve of pERK modulation was consistent at 1 and 6 hours in sNF96.2 and MeWo tumor cell lines. In NCI-1838, the increased pERK at lower concentrations was observed at 1 hour but not at 6 hours. Tissue and subtype specificity along with additional and differential mutations and mutational patterns within NF-1 may influence and add complexity to MAPK/ERK downstream signaling (32, 33).

In both the *in vitro* and *in vivo* models used in this study, an increase in pERK at low concentrations of tovorafenib was not associated with an increase in proliferation of cells or tumor growth. However, other studies (29) have observed an increase in tumor volume in a GEMM model of plexiform neurofibroma harboring germline *NF1-*LOF mutations following tovorafenib treatment. We propose that the biological significance of these observations is two-fold: (i) tovorafenib alone is insufficient to prevent tumor growth in the *NF1-*LOF setting and (ii) in the *NF1-*LOF setting, there is a potential risk of increased tumor growth in response to tovorafenib, as indicated by the *NF1-*LOF GEMM data (29). Our future goal is to explore more effective therapeutic options for pediatric patients with *NF1-*LOF tumors, focusing on treatments that could have a meaningful clinical impact, while avoiding the potential risk of paradoxical activation associated with tovorafenib as monotherapy.

The bell-shaped pERK modulation in *NF1-*LOF tumor cell lines seems to be a class effect. Type II RAF inhibitors, including TAK-632, belvarafenib, LXH254, and BGB-283 also showed a similar effect of increased pERK at lower concentrations and pERK inhibition at higher concentrations. Many type II RAF inhibitors, including tovorafenib, potently inhibit wild-type and altered BRAF and CRAF but are ARAF sparing, which may explain the pattern of pERK modulation observed in the *NF1-*LOF tumor cell lines treated with these agents. These results are consistent with findings from other studies that tovorafenib is ARAF sparing (34). RAF inhibitors are known to drive RAF dimerization, including BRAF::CRAF heterodimers and CRAF::CRAF homodimers, and there are more recent reports suggesting that ARAF heterodimerization may also occur. Most KRAS- or NRAS-mutant tumors have been shown to depend on CRAF, but not ARAF or BRAF, for growth. Recent articles have shown that CRAF can dimerize with ARAF more frequently than with BRAF in RAS-mutant tumors (35). Interestingly, the heterodimers can exhibit different sensitivity to type II RAF inhibitors, and ARAF depletion sensitized KRAS-mutant tumors cells to pan-RAF inhibition (36). It is possible that ARAF heterodimerization with CRAF may also occur in *NF1-*LOF mutant tumors, and ARAF sparing, type II RAF inhibitors may be insufficient to block all RAF activity in these tumors.

As tovorafenib is ARAF sparing, it does not inhibit heterodimers of BRAF:ARAF or CRAF:ARAF at lower concentrations. However, at higher concentrations, tovorafenib may be effective in inhibiting BRAF or CRAF within the heterodimers. Therefore, at lower concentrations, increases in pERK levels may reflect a relief of negative feedback as evident by the recently reported bell-shaped curve of pERK inhibition with LXH254 in ARAF-only–expressing RAS-mutant cells, in which pERK inhibition was observed only at higher concentrations (37). Although ARAF mutations were not observed in the tumor cell lines in our study, either ARAF heterodimerization or the emergence of ARAF mutations may drive resistance to type II RAF inhibitors, such as tovorafenib in *NF1-*LOF mutant or RAS-mutant tumors. Vertical pathway inhibition has been well established in overcoming resistance to single-agent BRAF inhibitors in melanoma harboring *BRAF *V600 mutations and thus driving deeper and more durable responses (38). Currently, the combination of BRAF and MEK inhibitors is the standard of care in *BRAF *V600E-mutated melanomas with three FDA approvals (39). In this study, single-agent tovorafenib had little anti-proliferative effect in *NF1-*LOF tumor cell lines, yet the combination of pimasertib with either tovorafenib or TAK-632 exhibited synergy in *NF1-*LOF tumor models *in vitro*. This is consistent with published data showing that preclinically the combination of type II RAF inhibitors and MEK inhibitors is synergistic in colorectal, melanoma, and lung tumor cell lines with mutations in NF1, BRAF, or KRAS (27, 40, 41). The combination of BGB-283, a type II RAF inhibitor, and selumetinib has been shown to be efficacious in *KRAS*-mutant tumors preclinically, and BGB-283 when used in combination with either trametinib or binimetinib was shown to prevent and overcome acquired resistance in *KRAS-*, *NRAS-*, *NF1-*, *BRAF*-mutated tumors (42, 43). Additionally, the combination of belvarafenib, a type II RAF inhibitor, with cobimetinib has been demonstrated to delay acquired resistance mediated by ARAF mutations in NRAS-mutant tumor cells preclinically, and in the clinic, this combination exhibited acceptable tolerability and encouraging efficacy in patients with NRAS-mutant melanoma (44, 45). Currently, selumetinib is the only drug approved for neurofibromatosis type 1 (46). Deeper and more durable responses may potentially be obtained by combining MEK inhibitors with a type II RAF inhibitor, such as tovorafenib in tumors with *NF1-*LOF.

In conclusion, tovorafenib monotherapy seems to be insufficient for the treatment of *NF1-*LOF tumors. This lack of efficacy may be attributed to the impact of tovorafenib on pERK because of its sparing of ARAF. Our data strongly support the combination of tovorafenib with a MEK inhibitor such as pimasertib as an effective strategy that may affect growth of tumors harboring *NF1-*LOF mutations, potentially leading to more durable responses.

## Supplementary Material

Fig S1 Supplementary Fig S1 - Body weights of mice treated with tovorafenib

Fig S2 Supplementary Fig S2 - Plasma PK exposure of tovorafenib in PDX-bearing and non-tumor bearing mice

Fig S3 Supplementary Fig S3 - Inhibition of proliferation by RAF inhibitors in NF1-LOF or BRAF mutant cell lines by RAF inhibitors

Fig S4 Supplementary Fig S4 - Combination of type II RAF inhibitors plus pimasertib in NF1-LOF tumor models

Table S1 Supplementary Table S1 - Tumor cell lines

Table S2 Supplementary Table S2 - PK parameters in mouse and human

Table S3 Supplementary Table S3 - Test agents
