# Supplementary material for: Preclinical Activity of the Type II RAF Inhibitor Tovorafenib in Tumor Models Harboring Either a BRAF Fusion or an NF1 Loss-of-Function Mutation
Source: Cancer Res Commun. 2025 Apr 23;5(4):668–79. doi: 10.1158/2767-9764.CRC-24-0451 (PMC12015663; doi:10.1158/2767-9764.CRC-24-0451)
Supplement: Fig S1 — Supplementary Fig S1 - Body weights of mice treated with tovorafenib [file crc-24-0451_fig_s1_suppsf1.docx]

**Supplementary Figure S1**: Body weights of mice treated with tovorafenib


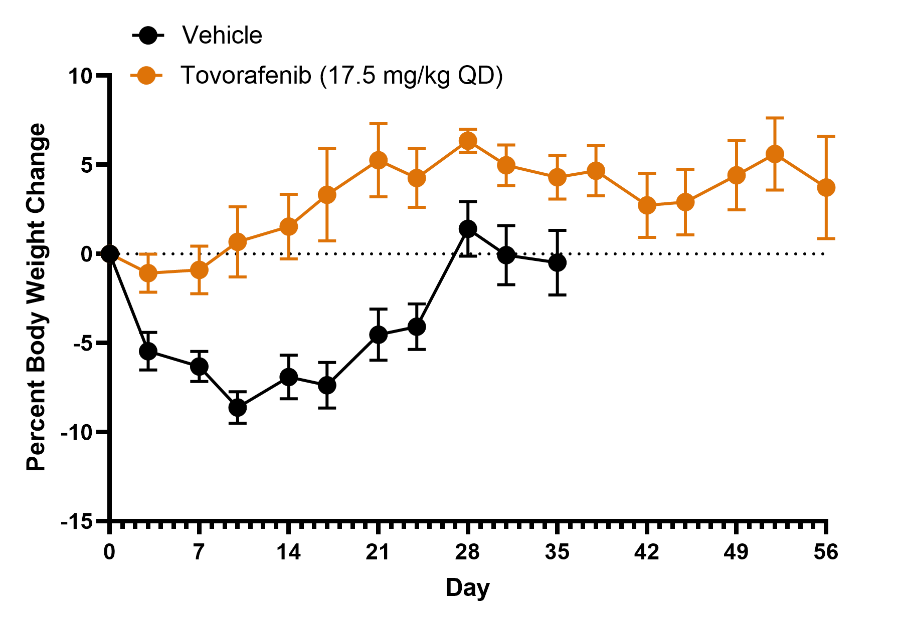


**A**

**B**


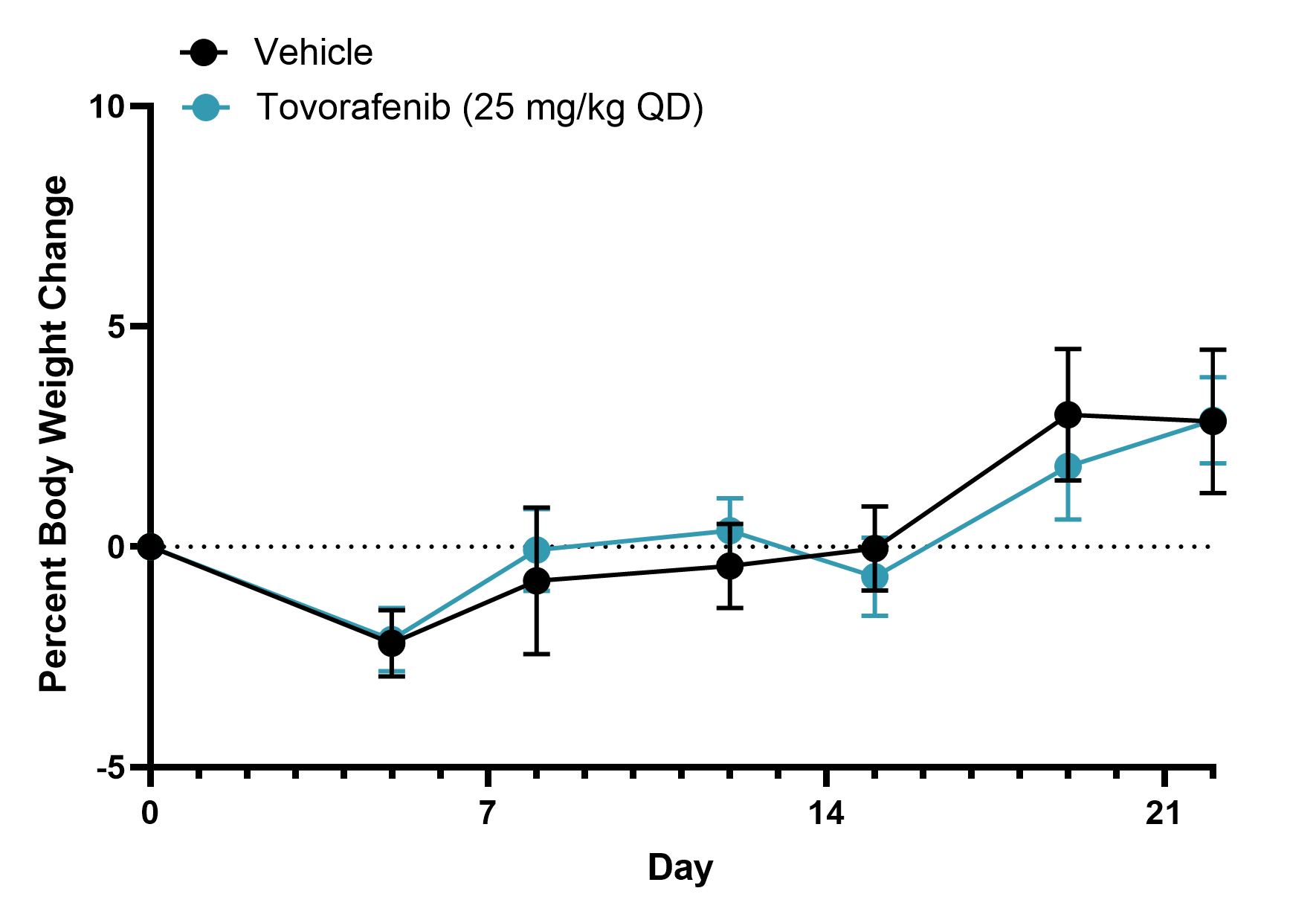


Body weight graphs for mice bearing *AGK*::*BRAF* fusion melanoma tumors treated orally QD with vehicle or tovorafenib at doses of (A) 25 mg/kg QD and (B) 17.5 mg/kg QD. Graphs were generated using GraphPad Prism software analysis.

QD, once a day.
