# Supplementary material for: Preclinical Activity of the Type II RAF Inhibitor Tovorafenib in Tumor Models Harboring Either a BRAF Fusion or an NF1 Loss-of-Function Mutation
Source: Cancer Res Commun. 2025 Apr 23;5(4):668–79. doi: 10.1158/2767-9764.CRC-24-0451 (PMC12015663; doi:10.1158/2767-9764.CRC-24-0451)
Supplement: Fig S2 — Supplementary Fig S2 - Plasma PK exposure of tovorafenib in PDX-bearing and non-tumor bearing mice [file crc-24-0451_fig_s2_suppsf2.docx]

**Supplementary Figure S2**: Plasma PK exposure of tovorafenib in PDX-bearing and non-tumor bearing mice


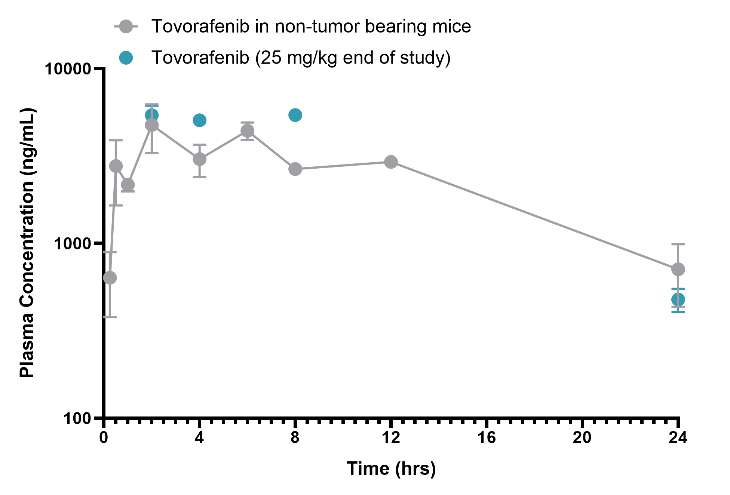


Plasma PK exposure graph for tovorafenib-dosed mice (female Nu/Nude mice; 25 mg/kg end of study) compared with tovorafenib in non-tumor bearing mice. Both the exposures and Ctrough were clinically relevant and at steady state. Graphs were generated using GraphPad Prism software analysis.

PDX, patient-derived xenograft; PK, pharmacokinetic.
