# Supplementary material for: Preclinical Activity of the Type II RAF Inhibitor Tovorafenib in Tumor Models Harboring Either a BRAF Fusion or an NF1 Loss-of-Function Mutation
Source: Cancer Res Commun. 2025 Apr 23;5(4):668–79. doi: 10.1158/2767-9764.CRC-24-0451 (PMC12015663; doi:10.1158/2767-9764.CRC-24-0451)
Supplement: Fig S3 — Supplementary Fig S3 - Inhibition of proliferation by RAF inhibitors in NF1-LOF or BRAF mutant cell lines by RAF inhibitors [file crc-24-0451_fig_s3_suppsf3.docx]

**Supplementary Figure S3**: Inhibition of proliferation by RAF inhibitors in *NF1-LOF* or *BRAF* mutant cell lines by RAF inhibitors


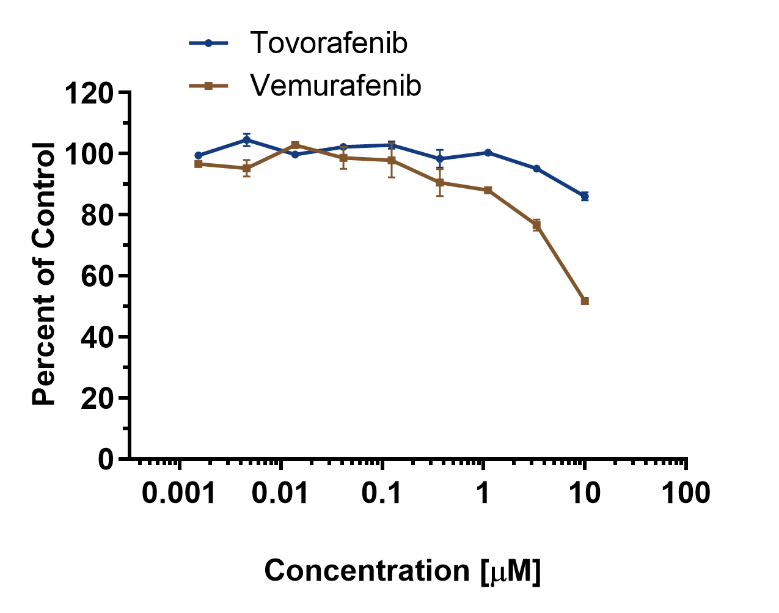

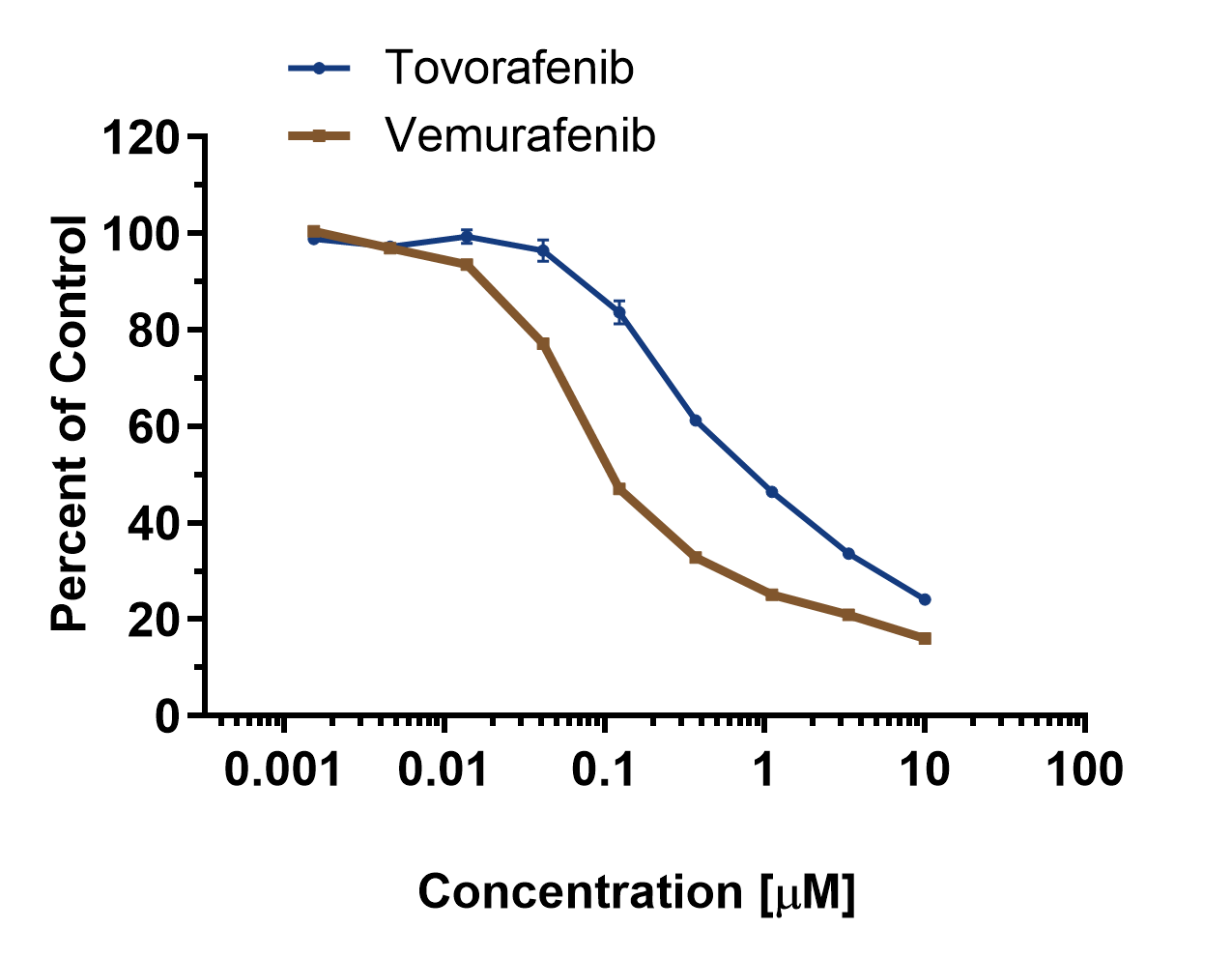

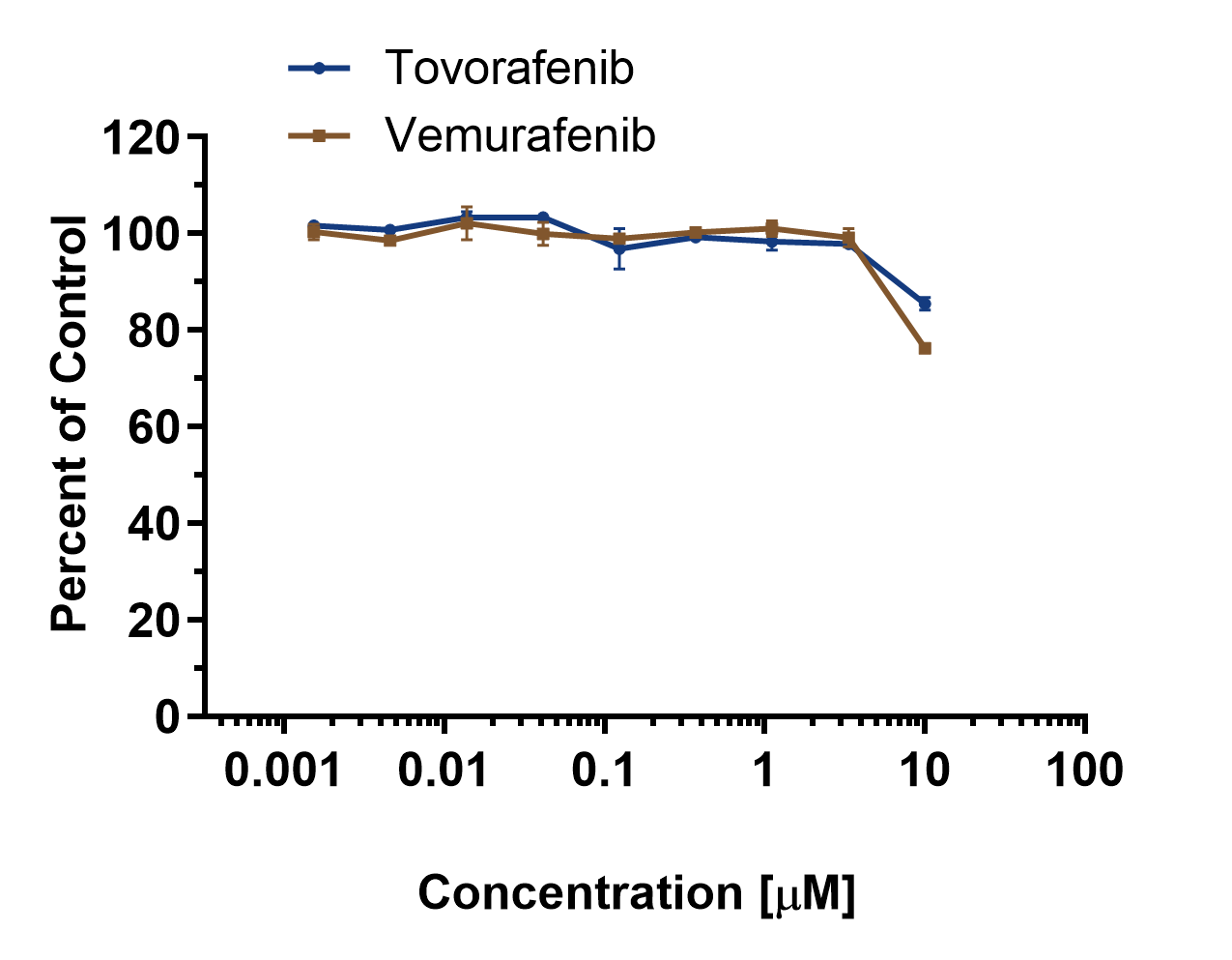

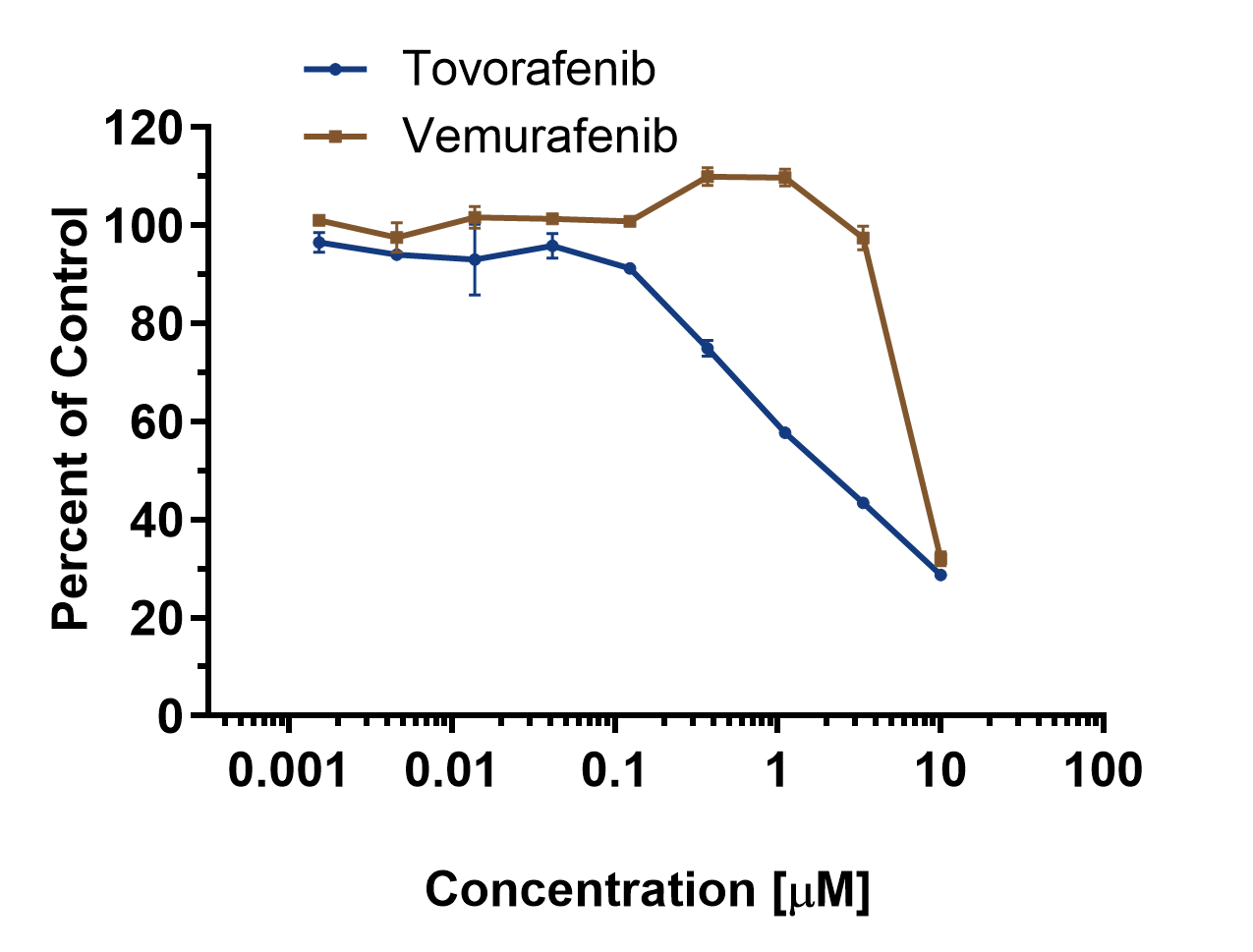


**A**

**C**

**B**

**D**

Proliferation was measured using CTG assay 72-hour post-treatment with either tovorafenib or vemurafenib in tumor cell lines harboring either an *NF1*-LOF mutation: (A) sNF96.2, (B) MeWo, or (C) NCI-H1838, or a BRAF V600E mutation: (D) A375. Concentration response curves were generated using GraphPad Prism software analysis.

NF1-LOF, neurofibromin 1 loss of function.
