# Supplementary material for: Preclinical Activity of the Type II RAF Inhibitor Tovorafenib in Tumor Models Harboring Either a BRAF Fusion or an NF1 Loss-of-Function Mutation
Source: Cancer Res Commun. 2025 Apr 23;5(4):668–79. doi: 10.1158/2767-9764.CRC-24-0451 (PMC12015663; doi:10.1158/2767-9764.CRC-24-0451)
Supplement: Fig S4 — Supplementary Fig S4 - Combination of type II RAF inhibitors plus pimasertib in NF1-LOF tumor models [file crc-24-0451_fig_s4_suppsf4.docx]

**Supplementary Figure S4**: Combination of type II RAF inhibitors plus pimasertib in *NF1*-LOF tumor models

**
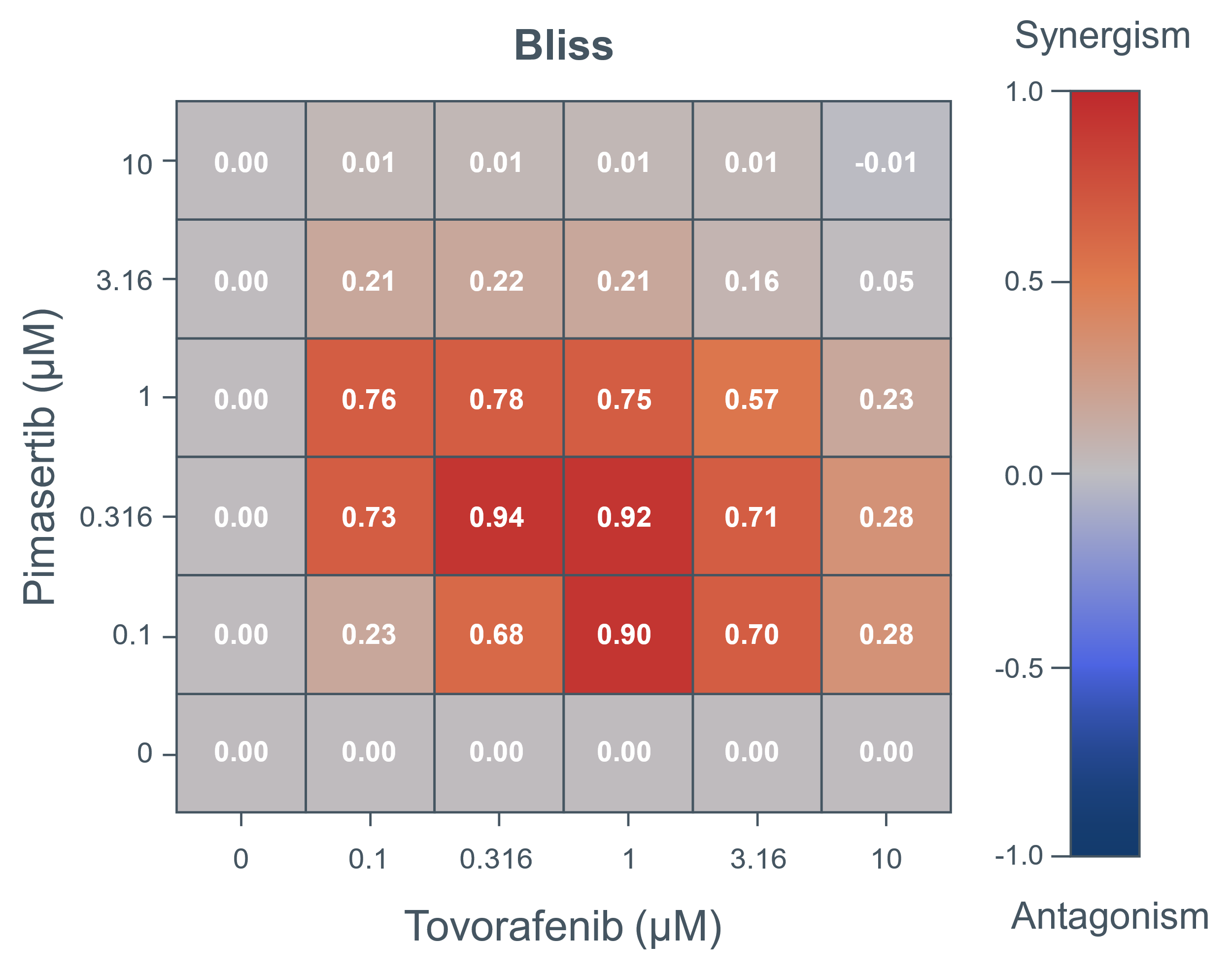

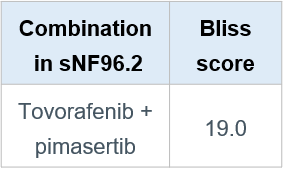
**

**A**

*Bliss score >0=synergy*

**
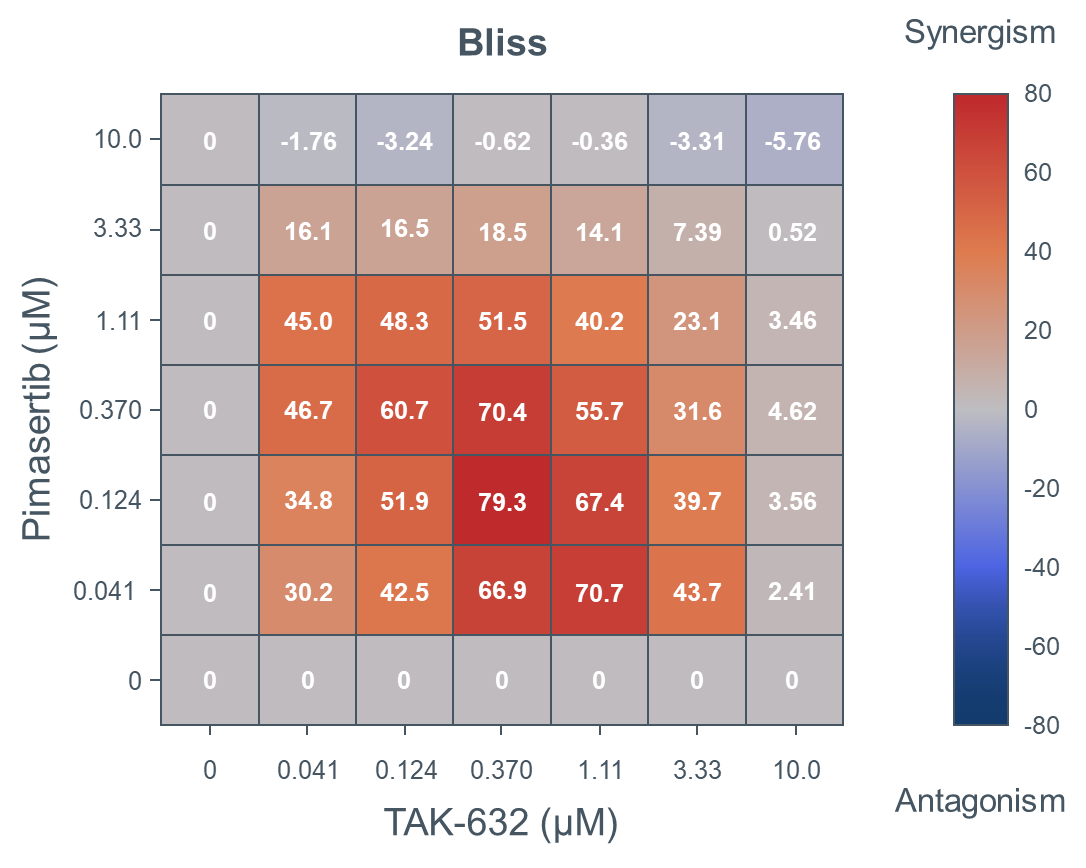
**

*Bliss score >10=synergy*

**B**

**
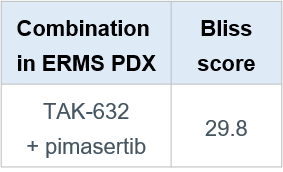
**

(A) In sNF96.2 cells, synergy was assessed 72 hours post-treatment with tovorafenib plus pimasertib in a 5×5 matrix combination format in 2D, followed by Bliss independence analysis. Pimasertib was added once at the start of the experiment and for tovorafenib, repeated application occurred on days 2 and 3. Positive values (Bliss Index ≥0.15, red) indicate synergy, negative values (Bliss Index ≤-0.15, blue) indicate antagonism, and 0 is neutral indicating an additive effect. (B) ERMS PDX model tumor cells were isolated from the PDX tumor fragments and cultured *ex vivo* to assess synergy after 144 hours of treatment with TAK-632 plus pimasertib in a 6×6 matrix combination format in 3D, followed by Bliss independence analysis. Positive values (Bliss Index >10, red) indicate synergy, negative values (Bliss Index <10, blue) indicate antagonism, and Bliss Index between -10 to 10 indicate an additive effect. Due to the 3D format of the assay, conditions were not amenable for repeated application of tovorafenib, thus TAK-632 was used as a positive type II RAF inhibitor control. These experiments were performed once.

ERMS, embryonal rhabdomyosarcoma; NF1-LOF, neurofibromin 1 loss of function; PDX, patient-derived xenograft.
