# Supplementary material for: Preclinical Activity of the Type II RAF Inhibitor Tovorafenib in Tumor Models Harboring Either a BRAF Fusion or an NF1 Loss-of-Function Mutation
Source: Cancer Res Commun. 2025 Apr 23;5(4):668–79. doi: 10.1158/2767-9764.CRC-24-0451 (PMC12015663; doi:10.1158/2767-9764.CRC-24-0451)
Supplement: Table S1 — Supplementary Table S1 - Tumor cell lines [file crc-24-0451_table_s1_suppst1.docx]

**Supplementary Table S1:** Tumor cell lines

| **Tumor cell line** | **Mutation** | **Tissue type** | **Vendor (Cat. No.)​** | **RRID** | **Culture medium​** |
| --- | --- | --- | --- | --- | --- |
| sNF96.2​ | *NF1*-LOF | MPNST | ATCC (CRL-2884) | CVCL_K281 | DMEM + 10% FBS |
| MeWo​ | *NF1*-LOF | Melanoma | ATCC (HTB-65) | CVCL_0445 | EMEM + 10% FBS |
| NCI-H1838 | *NF1*-LOF | Lung | ATCC (CRL-5899) | CVCL_1499 | RPMI-1640 + 10% FBS |
| A375​ | BRAF V600E | Melanoma | ATCC (CRL-1619) | CVCL_0132 | DMEM + 10% FBS |
| ERMS PDX  *(ex vivo)* | *NF1*-LOF | ERMS | Champions Oncology (N/A) | N/A | DMEM high glucose with 10% FBS, antibiotic, antimycotic, 1:500 Primocin antimicrobial agent, and 1:100 GlutaMAX |

Culture media DMEM and RPMI-1640 were purchased from Gibco (Cat. No. 11965-092 and 22400-089, respectively) and EMEM was purchased from ATCC (Cat. No. 30-2003). GlutaMAX was purchased from Gibco (Cat. No. 35050-061), Primocin was purchased from InvivoGen (Cat. No. ant-pm-1), and antibiotic-antimycotic was purchased from Invitrogen (Cat. No. 15240-096).
DMEM, Dulbecco's Modified Eagle Medium; ERMS, embryonal rhabdomyosarcoma; FBS, fetal bovine serum*;* MPNST, malignant peripheral nerve sheath tumour; *NF1*-LOF, neurofibromin 1 loss of function; PDX, patient-derived xenograft; RRID, Research Resource Identifiers; RPMI, Roswell Park Memorial Institute.
