# Supplementary material for: Preclinical Activity of the Type II RAF Inhibitor Tovorafenib in Tumor Models Harboring Either a BRAF Fusion or an NF1 Loss-of-Function Mutation
Source: Cancer Res Commun. 2025 Apr 23;5(4):668–79. doi: 10.1158/2767-9764.CRC-24-0451 (PMC12015663; doi:10.1158/2767-9764.CRC-24-0451)
Supplement: Table S2 — Supplementary Table S2 - PK parameters in mouse and human [file crc-24-0451_table_s2_suppst2.docx]

**Supplementary Table S2**: PK parameters in mouse and human

| **Species** | **Dose Regimen** | **PK Parameter** | |
| --- | --- | --- | --- |
|  |  | **Daily AUC_0-24_ (ng*hr/mL)** | **C_min_ (ng/mL)** |
| **Mouse PK**  **(at efficacious doses)** | 12 – 25 mg/kg, QD | 50,100 – 96,600 | 596 – 1265 |
| **Human PK** | 600 mg, QW | 47,600^a^ | 899 |

| ^a^: Calculated from weekly AUC_0-168_ divided by 7. |
| --- |

AUC, area under the curve; PK, pharmacokinetic; QD, once a day; QW, once a week.
