# Supplementary material for: Preclinical Activity of the Type II RAF Inhibitor Tovorafenib in Tumor Models Harboring Either a BRAF Fusion or an NF1 Loss-of-Function Mutation
Source: Cancer Res Commun. 2025 Apr 23;5(4):668–79. doi: 10.1158/2767-9764.CRC-24-0451 (PMC12015663; doi:10.1158/2767-9764.CRC-24-0451)
Supplement: Table S3 — Supplementary Table S3 - Test agents [file crc-24-0451_table_s3_suppst3.docx]

**Supplementary Table S3**: Test agents

| **Inhibitor** | **Type of inhibitor** | **Storage** | **Purity, %**  **(appearance**^a^**)** | **Vendor**  **(Batch or Lot No.)​** |
| --- | --- | --- | --- | --- |
| Tovorafenib | Type II RAF | Ambient | 100.00 | Day One Biopharmaceuticals  (MA18-004, MA18-005) |
|  |  | 2–8°C | 99.00  (white to yellow powder) | Serán Bioscience  (DM-DA1-002-A)^b^ |
| TAK-632 |  | -20°C | 98.90 | Pacific Pharmaceutical Services  (PGS-89-120) |
| LXH254 |  | -20°C | 99.96 | Selleck  (GRN:104-1606-022) |
| Belvarafenib |  | -20°C | 96.58 | Med Chem Express  (63178) |
| BGB283 | dual RAF and EGFR | -20°C | 99.73 | Selleck  (GRN:104-1606-021) |
| Vemurafenib | Type I RAF | -20°C | 98.33 | Med Chem Express  (05724) |
| Pimasertib | MEK | Ambient | 99.90  (yellow solid) | Merck KGaA  (PGS-104-08, PGS-104-48) |
|  |  |  | Not specified | Day One Biopharmaceuticals  (PGS-1156a) |

All experiments conducted by Crown BioScience sourced tovorafenib and pimasertib from Serán Bioscience and Merck KGaA, respectively; all other experiments sourced tovorafenib and pimasertib from Pacific Pharmac. Services. All test agents were stored in a light protected, moisture free environment. ^a^Where specified; ^b^Tovorafenib purchased as a 40% HPMCAS-M spray-dried dispersion.
